# Supplementary material for: Vesicle and reaction-diffusion hybrid modeling with STEPS
Source: Commun Biol. 2024 May 15;7:573. doi: 10.1038/s42003-024-06276-5 (PMC11096338; doi:10.1038/s42003-024-06276-5)
Supplement: Supplementary file 2 — Supplementary Information [file 42003_2024_6276_MOESM2_ESM.pdf]

Vesicle and reaction-diffusion hybrid modeling with STEPS

Iain Hepburn[1], Jules Lallouette[1], Weiliang Chen[1], Andrew R. Gallimore[1], Sarah Y. Nagasawa-Soeda[1] and Erik De Schutter[1*]

[1]Computational Neuroscience Unit, Okinawa Institute of Science and Technology Graduate University, 1919-1 Tancha, Onna-son, Okinawa, Japan.

*Corresponding author(s). E-mail(s): erik@oist.jp

Supplementary Information

Supplementary Table 1 | Kinetic Processes in STEPS 5.0

Kinetic processes	Dep Locations: Triangles	Dep: Vesicle surface	Dep: Raft surface	Dep: Raft surface (anti-dep)	LHS Locations: Tetrahedrons	LHS: Triangles	LHS: Vesicle surface	LHS: Vesicle lumen	LHS: Raft surface	RHS Locations: Tetrahedrons	RHS: Triangles	RHS: Vesicle surface	RHS: Vesicle lumen	RHS: Raft surface
Volume reaction					[Species]					[Species]				
Surface reaction					[Species]	[Species]				[Species]	[Species]			
Vesicle Surface reaction		[Species]			[Species]	[Species] 1	[Species/Link Species]			[Species]	[Species]	[Species/Link Species]	[Species]	
Raft Surface reaction			[Species]	[Species]	[Species]	[Species]			[Species]	[Species]	[Species]			[Species]
Vesicle Binding		[Species/Link Species]					[Species] *					[Link Species] *		
Vesicle Unbinding							[Link Species] *					[Species] *		
Exocytosis		[Species]			[Vesicle] *		[Species] 2.1	[Species] 2.2		[Species] 2.2	[Species/Raft]			[Species] 2.1
Exocytosis (kiss-and-run)		[Species]			[Vesicle] *		[Species] 2.1	[Species] 2.2		[Species/Vesicle] 2.2		[Species]	[Species]	
Endocytosis						[Species] 3				[Vesicle] *		[Species] 3		
Raft endocytosis						[Raft] *			[Species]	[Vesicle] *		[Species]		
Raft generation						[Species] 4					[Raft] *			[Species] 4
Raft dissolution				[Species]		[Raft] *			[Species] 5		[Species] 5			

Color code: Species | Link Species | Vesicle | Raft

1: If species on triangles are involved, the reaction also requires a minimum distance (between the vesicle and the triangle) under which it can happen.

2.1, **2.2**: Upon exocytosis, species on the vesicle surface are added to the raft surface (if any) and species in the vesicle lumen are released into the outside tetrahedron.

3: Triangles are grouped in endocytic zones. During endocytosis, all species in the zone are assigned to the surface of the vesicle.

4: The species are removed from the triangle and assigned to the surface of the newly created raft.

5: The species from the surface of the raft that undergoes dissolution are assigned to the triangle from the center of the raft.

Supplementary Table. 1 | Summary of entities and locations involved in each kinetic process. The color code indicates which type of entity is involved while the different column groups indicate the role that entities can play in the corresponding location. The entries in the LHS Locations group indicate that the corresponding entities are consumed by the kinetic process. The entries in the RHS Locations group indicate that the entities are produced by the process. The entries in the Dep Locations group indicate that the entities are required for the process to happen but not consumed. The 4th column of this group indicated an anti-dependency, the kinetic process can be prevented from happening by the presence of an entity. A star (*) indicates that a role is mandatory, other roles are optional. Although this table shows all the possible entity roles for each kinetic process, they are rarely all used at the same time when declaring e.g. a specific Vesicle surface reaction.

Supplementary Note 1 | Vesicle diffusion

A single vesicle of diameter 50nm was injected into the centre of a tetrahedral mesh of a 10μm radius sphere. The vesicle was given a diffusion rate of $1\mu m^2 s^{-1}$ and the simulation was run for 1s, with the distance from the origin measured at 0.05s intervals. This simulation was repeated 10,000 times with the mean taken for the data shown in Fig. 3b (yellow). For the cytosolic occupancy simulations, tetrahedrons representing a total of 20%, 40% and 60% of the overall simulation volume were randomly removed from the simulation effectively creating cytosolic obstructions, for the same simulation protocol as above.

Supplementary Note 2 | Mossy Fibre Terminal simulations: how steric interactions affect vesicle mobility

To mimic the simulation conditions of Rothman et al [1], a tetrahedral mesh of the Mossy Fibre Terminal volume of cubic boundaries 3μm x 3μm x 7μm was created with 565,958 tetrahedrons. Cylindrical mitochondria of 0.3μm diameter and 2.25μm length on the z-axis were created at random positions within the volume up to a total volume occupancy of 28%, numbering 110 in total, creating fixed obstructions within the cytosolic volume.
For the simulation, two types of vesicles were used: mobile vesicles, making up 17% of the overall MFT volume, and immobile vesicles making up 25%. The mobile vesicles were given a diffusion coefficient of $0.06\mu m^2 s^{-1}$ and all vesicles had a diameter of 49nm. The initial state of the simulation is shown in the inset of Fig. 3c. The simulation was run for 0.3s and the mean square displacement of mobile vesicles starting within a 1μm radius of the mesh centre was recorded. This was divided by the time vector and 6 to give an apparent diffusion rate vs time (because $<R^2> = 6Dt$), as shown in Fig. 3c.

Supplementary Note 3 | Active transport

Active transport is simulated in STEPS on simulation objects called 'Paths' that represent cytoskeletal filaments such as actin or microtubules and may be branched. A user-defined stepsize and mean speed for vesicle active transport along these paths is then used to simulate fixed-sized steps from start to end. The time period until the next step ('dwelltime') is taken either from a single-exponential or double-exponential, which is calculated from the given parameters so that the total distance traveled divided by the summed dwelltimes equals the expected speed (with noise).

Supplementary Fig. 1 shows the sampled 1-step (single-exponential) and 2-step (double-exponential) distributions, and recorded distributions from a STEPS simulation. In this simulation, the vesicle travels along the path at a mean speed of $0.8\mu m.s^{-1}$ with a stepsize of 36nm, giving a mean dwelltime of 0.045s.

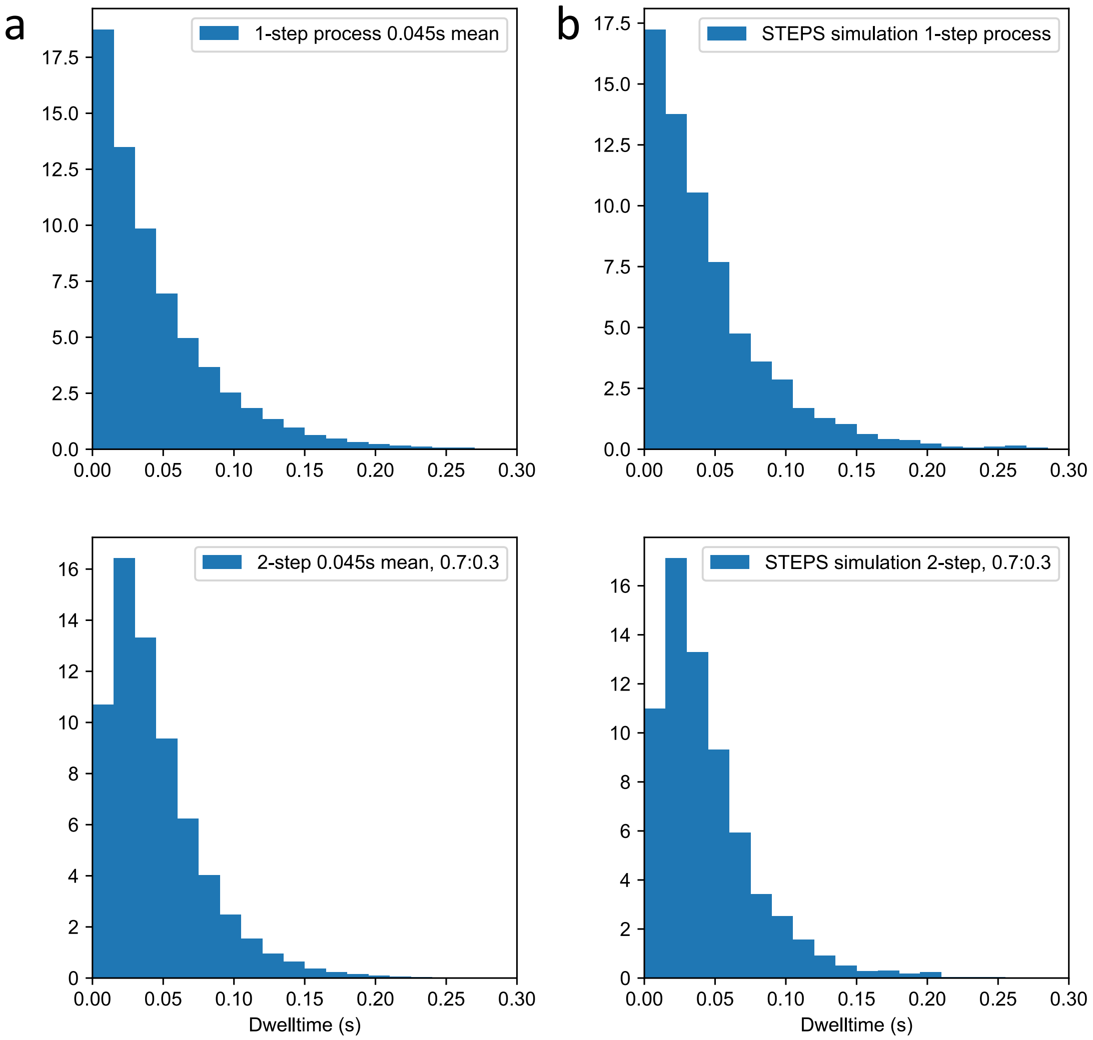

Supplementary Fig. 1 | a) Single-exponential (top panel) and double-exponential in 0.7:0.3 ratio (bottom panel) dwelltime, solved numerically. b) the recorded dwelltimes for a vesicle undergoing active transport on a path in a STEPS simulation, validating the implementation.

Supplementary Note 4 | Active transport validation

We created a model including two distinct paths with vesicles interacting by different speeds on each path in order to validate the active transport implementation in STEPS. The mean speeds were defined as $0.5\mu m.s^{-1}$ on the 1st path and $0.3\mu m.s^{-1}$ on the 2nd path, with a small step size of just 0.1nm to counteract stochastic effects for the purposes of validation (which gives an expected mean dwelltime of 0.2ms and 0.33ms respectively). The effective speed of a vesicle on the path is then equal to the distance travelled divided by the summed dwelltimes. Vesicles will optionally only interact with a path if a given species dependency is met, and this was utilized in the model to ensure vesicles interacted with the expected path at the expected time. Vesicles

were injected at the start of the paths at 2s intermittent time intervals starting at 0s on the 1st path and 0.8s on the 2nd path. Both paths were on the z-axis, and the z-position of the vesicle was recorded and compared to the expected position based on the speed as shown in Fig. 3d.

Supplementary Note 5 | Vesicle surface molecule diffusion

To perform our own validation of the approximate propagator algorithm of Ghosh et al [2] for surface diffusion on vesicles, as implemented in STEPS, we designed a model in which surface diffusion on a spherical mesh could be compared directly to output from the algorithm. We created a tetrahedral mesh of a sphere of radius 50nm and simulated diffusion on the surface in STEPS, simulating 2D diffusion between the edges of surface triangles. The surface diffusing species was given a diffusion coefficient of $1\mu m^2 s^{-1}$ and the model was simulated for 3ms 100,000 times each time with a different random number sequence. The angular displacement distribution was then compared to the Ghosh algorithm at 2ms, as shown in Fig. 3e, and the angular displacement mean and standard deviation at 0.1ms intervals were compared over the whole 3ms simulation as shown in Fig. 3f. Supplementary Fig. 2 shows the full distribution also at 1ms (a) and 3ms (b), and compares the Ghosh algorithm to the STEPS implementation (c).

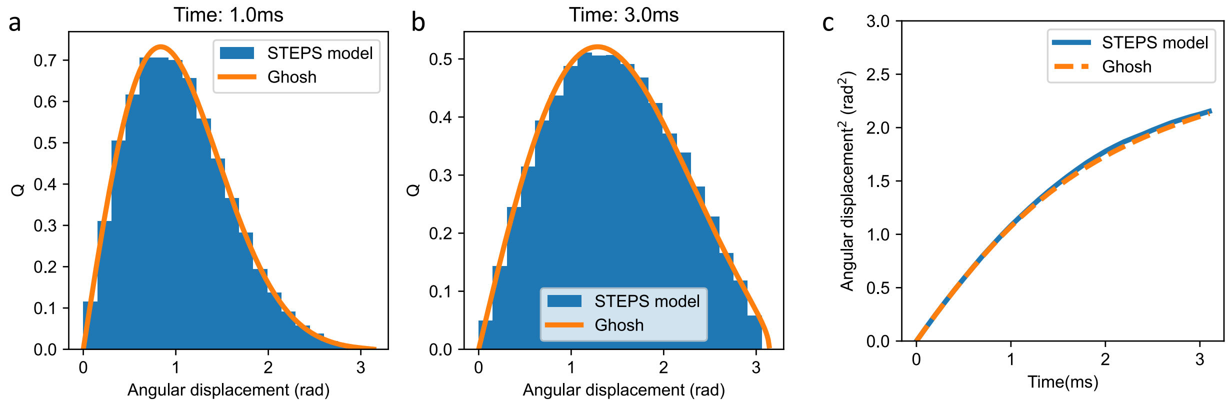

Supplementary Fig. 2 | The STEPS model of surface diffusion on a sphere compared to the Ghosh approximate propagator algorithm. a) angular displacement distribution at 1ms and b) 2ms. c) The STEPS surface diffusion model (blue) and Ghosh algorithm (orange) are compared as mean output.

Supplementary Note 6 | Exocytosis

To validate the implementation of exocytosis with the SSA, we created a model in which 10 vesicles of 40nm diameter were free to diffuse around a spherical volume of 0.5μm diameter at a diffusion rate of $1\mu m^2 s^{-1}$. Exocytosis could then occur anywhere on the membrane at a rate, k, of 5 s^{-1}. Under these conditions, diffusion is not rate limiting and with exocytosis implemented as a first-order reaction the vesicle population should follow the expected exponential decay:

$$N(t) = N_0 e^{-kt} \quad (1)$$

And the standard deviation also has a known analytical solution:

$$\sigma(t) = \sqrt{N_0 e^{-kt}(1 - e^{-kt})} \quad (2)$$

We simulated the exocytosis model for 0.2s and compared the mean and standard deviation of 1000 runs to Supplementary Equations 1 and 2, as shown in Fig. 4a.

Supplementary Note 7 | Endocytosis

We used two models to validate Endocytosis in STEPS. In the first model, endocytosis occurred from membrane Rafts. On the surface of a mesh of a sphere of 0.5μm diameter, 20 immobile rafts were randomly placed and specified to undergo endocytosis at a rate of 20 s^{-1}. The resulting raft number on the surface is then expected to follow an exponential decay following Supplementary Equations 1 and 2, and we ran the simulation 1000 times with different random number sequences to make the comparison to the analytical solution, as shown in Fig. 4b.

In the second model the alternative mode of endocytosis was tested, occurring when a certain species signature is met within surface mesh triangles. In this simple test model, a dependency of just 1 molecule was used with endocytosis occurring at a rate of 10 s^{-1}, and with 10 molecules randomly placed on the mesh surface. Since the dependent species is absorbed during endocytosis and becomes located on the surface of the endocytosed vesicle, the dependent species population in the mesh surface is also expected to follow an exponential decay following Supplementary Equations 1 and 2, with the mean and standard deviation of 1000 simulations (each with a different random number sequence) shown in in Fig. 4c. For this model, the vesicle number is also shown in Fig. 4d, which is expected to rise as:

$$N(t) = N_0 + M_0(1 - e^{-kt}) \quad (3)$$

where N is the vesicle number (in this model $N_0 = 0$, the vesicle number is 0 at time 0) and M_0 is the initial dependent species number.

Supplementary Note 8 | Vesicle surface molecule interactions

To validate the implementation of vesicle interactions with their environment through molecules in their surface, we tested 4 different types of reactions occurring on 10 vesicles of diameter 40nm freely diffusing around a spherical volume of 0.5μm diameter at a rate of 1$\mu m^2 s^{-1}$:

- A first-order irreversible reaction: $A \xrightarrow{k} B$, following Supplementary Equations 1 and 2 and shown in Fig. 5a. For this model, a total of 100 molecules were injected (10 per vesicle) and allowed to decay at a rate of 10 s^{-1}. The simulation was run 1000 times each time with a different random number sequence.

In addition, Supplementary Figure 3 shows the simulated rate of the first order irreversible reaction validation model compared to the expected rate, as a percentage error, for different meshes. These meshes contained average tetrahedron sizes from 120nm to 12.8nm. As expected, error is low (less than 0.5%) in every case and there is no effect of mesh size on the simulated reaction rate for this reaction.

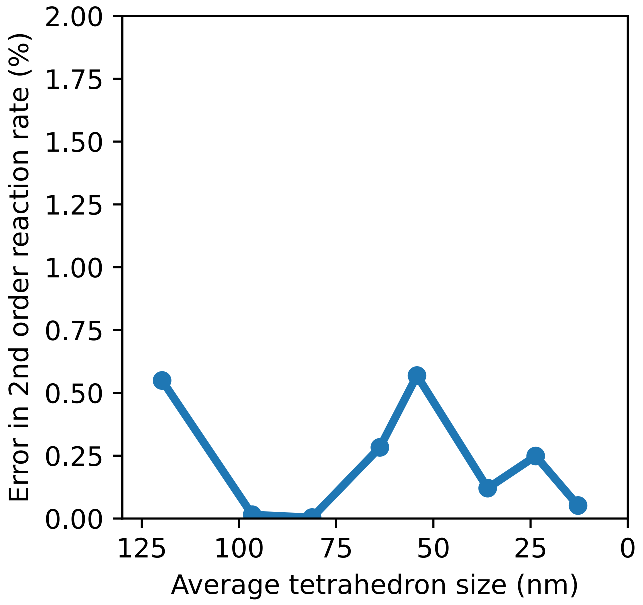

Supplementary Fig. 3 | The error in simulated first order irreversible vesicle surface reaction rate of for meshes of different average tetrahedron size. The rate is calculated from fitting Supplementary Equation 1 to the mean data of n=1000 independent simulation each with different random number sequences, for each different mesh.

- A first-order reversible reaction: $A \underset{k_b}{\overset{k_f}{\rightleftarrows}} B$

If $[B]_0$=0 for simplicity, the expected equilibrium concentrations are:

$$[A]_{eq} = \frac{k_b[A]_0}{k_f+k_b} \tag{4}$$

$$[B]_{eq} = [A]_0 - [A]_{eq} \tag{5}$$

Where [ ] indicates cytosolic concentrations. The reversible reaction was implemented on the vesicle surface with 500 molecules of A per vesicle surface (which is quite high to reduce the effects of noise) giving $[A]_0$ = 135.6μM with $[B]_0$ = 0μM, and with k_f=100s^{-1} and k_b=20s^{-1}. The mean cytosolic concentrations over 10 runs are shown in Fig. 5b where it can be seen that they approach the expected equilibrium concentrations.

- A second-order irreversible reaction: $A + B \overset{k}{\rightarrow} C$

If $[A]_0$ = $[B]_0$ then:

$$\frac{1}{[A]} = kt + \frac{1}{[A]_0} \quad (6)$$

and so it is expect that a plot of 1/[A] vs time will have slope k and intercept $1/[A]_0$.

This model was implemented in STEPS with species A located in vesicle surfaces, and species B in the cytosol. The species interact through the second order irreversible reaction with an initial count of 1000 each (100 molecules of species A for each of the 10 vesicles, and 1000 molecules of B in the cytosol). The reaction rate was $2\mu M^{-1}s^{-1}$ and the simulation was run 1000 times each time with a different random number sequence. Fig. 5c compares output from STEPS to the analytical solution of Supplementary Equation 6.

The above is for the special case where the initial (and resulting) concentration of A and B are equal to each other, but if that is not the case then:

$$ln\left(\frac{[B]}{[A]}\right) = ln\left(\frac{[B]_0}{[A]_0}\right) - ([A]_0 - [B]_0)kt \quad (7)$$

For this test, the vesicle surface species, A, was given a starting count of 500 (50 per vesicle) and the cytosolic species, B, was given a starting count of 250 and they were allowed to react with each other at a rate of $0.5\mu M^{-1}s^{-1}$, with the simulation run 1000 times each time with different random number sequences. Fig. 5d shows the output of the STEPS simulation compared to the analytical solution of Supplementary Equation 7.

Supplementary Note 9 | Vesicle binding reactions

In the validation model of the binding interactions between vesicles (and formation of 'link' species), because both reactants are of the same species their initial concentrations are identical and we can use Supplementary Equation 6. 100 small vesicles of 10 nm diameter and with 1 binding species in each vesicle surface were injected into the cytosolic volume of a spherical mesh of $0.5\mu M$ diameter. The binding interaction then took place with a reaction rate of $2\mu M^{-1}s^{-1}$ and the simulation was run 100 times with different random number sequences. Fig. 5e shows the output of this model to the analytical solution of Supplementary Equation 6.

Supplementary Note 10 | Raft diffusion

The 2D solution to the diffusion equation from a point source is:

$$C(r,t) = \frac{N}{4\pi Dt} e^{\left(-r^2/_{4Dt}\right)} \quad (8)$$

where C is the density (i.e. the number of molecules per unit area), N is the total number of molecules, D is the diffusion rate, and r is the distance from source.

The simplest test of raft diffusion in STEPS is to test conditions that approximate the point source by injecting rafts initially in an area that is small compared to the range of diffusion. We created a mesh of a flat circular disk shape with a radius of $10\mu m$ and injected rafts of 10nm radius close to the center of one of the circular faces, then allowed them to diffuse at a rate of

$10\mu m^2 s^{-1}$. Overall, 100 rafts were injected. The density of rafts on the circular face was then compared to the analytical solution of Supplementary Equation 8 at times 30ms, 50ms, 70ms, 90ms and 110ms, as shown in Fig. 6a, as the mean of 1000 runs of the simulation with different random number sequences. Boundary effects were negligible because very few rafts reached the 10μm circular edge at these times.

Supplementary Note 11 | Raft molecule reactions

Reactions involving raft surface molecules were tested quite similarly as for section 9, with the difference that the reactants were situated on rafts undergoing diffusion on the mesh surface, and with some variation in parameters. 10 rafts of 100 nm diameter were placed randomly on the surface of a mesh of a 0.5μm diameter sphere and allowed to diffuse at a rate of $1\mu m^2 s^{-1}$.

Firstly, first order irreversible reactions were tested against Supplementary Equations 1 and 2, as shown in Fig. 6b. This model was initialized with 50 molecules (5 per raft) which decayed by a rate of $5s^{-1}$. Fig. 6b displays the output of this model against Supplementary Equations 1 and 2 for 500 runs of the simulation with different random number sequences. For the first order reversible interactions, 1000 molecules were initialised per raft with a forward rate constant of $100s^{-1}$ and backward rate constant of $20s^{-1}$. Fig. 6c shows the mean output of this model against Supplementary Equations 4 and 5 for the equilibrium concentrations, over 10 runs of the simulation with different random number sequences.

Rafts themselves in STEPS can undergo special types of first order reactions that can generate or degrade rafts and can be tested in a production and degradation model. For simultaneous degradation and production such as: $A \xrightarrow{k_1} \emptyset$, $\emptyset \xrightarrow{k_2} A$
the stationary distribution is:

$$\Phi(n) = \frac{1}{n!}\left(\frac{k_2}{k_1}\right)^n e^{-\left(k_2/k_1\right)} \tag{9}$$

We implemented a model that could be compared to Supplementary Equation 9, with a raft generation reaction representing production and a raft dissociation reaction representing degradation. Rafts were generated from a species signature of just 1 molecule, with the number of these molecules in the mesh surface clamped at 1 throughout the simulation. This gave steady production of rafts, at a rate of $10s^{-1}$. Degradation of rafts also occurred at a species signature of 1 molecule in the raft, at a rate of $1s^{-1}$. The resulting number of rafts is then expected to follow Supplementary Equation 9. Fig. 6d compares the output of the STEPS simulation to the analytical solution for a long simulation time of 100,000s (with samples taken every 0.1s) in order to negate any sampling error.

For the interactions between rafts and cytosolic species, we implemented second order reactions that could be comparted to Supplementary Equations 6 and 7. In the first test, 1000 raft surface species (100 per raft) could react with 1000 cytosolic species at a rate of $2\mu M^{-1}s^{-1}$. Fig. 6e compares the output of the STEPS simulation (mean of 500 runs) to Supplementary

Equation 6 for this model. In the second test, 500 raft surface species (50 per raft) reacted with 250 cytosolic species at a rate of $1\mu M^{-1}s^{-1}$, with the output from STEPS (mean of 500 runs) compared to Supplementary Equation 7, as shown in Fig. 6f.

Supplementary Note 12 | Vesicle occupancy effect on distribution of regular SSA species

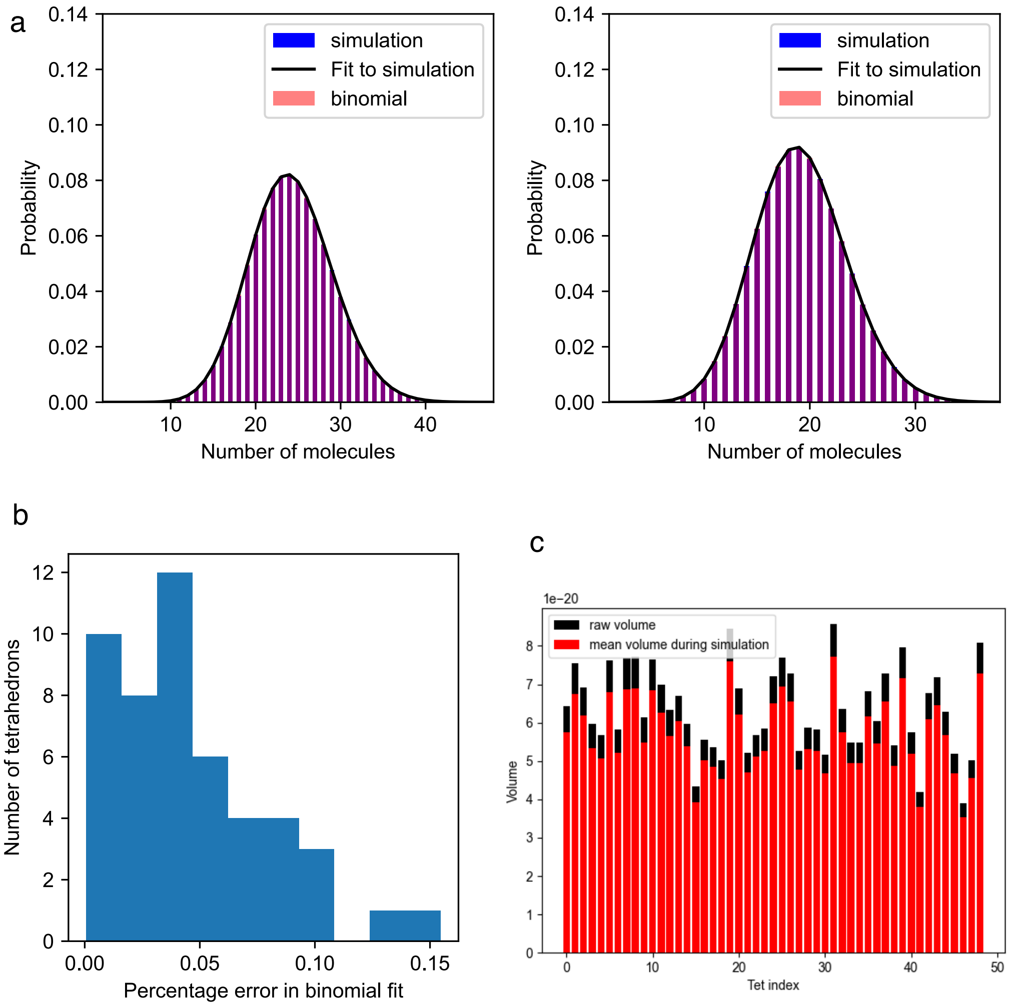

Supplementary Fig. 4 | Distribution of diffusing molecules over time in a model with 10% vesicle occupancy. a) The distribution of molecules in two example tetrahedrons in the mesh (blue bars) and binomial fit to the data (black line) compared to the expected binomial distribution (red bars). b) Percentage error in the binomial fit for all 49 tetrahedrons in the mesh. c) The recorded tetrahedron volume with vesicle occupancy subtracted (red) with the top of the bars showing the unoccupied volume, for all 49 tetrahedrons in the mesh. The region in black therefore shows the mean volume occupied by vesicles throughout the simulation.

Because the presence of vesicles can affect the diffusion of regular SSA species by a volume occupancy effect (as detailed in Methods) we tested the distribution of cytosolic species in a model with 10% occupancy by vesicles. We created a mesh of a spherical volume of a radius $10\mu m$ but of just 49 tetrahedrons for analytical purposes. Vesicles of diameter 50nm were

inserted into the volume, comprising 4728 vesicles in total to give 10% occupancy, and they diffused at a rate of $1\mu m^2 s^{-1}$. 1000 molecules were also injected into the volume and diffused at a rate of $10\mu m^2 s^{-1}$. The simulation was run for 100s with the population of species recorded per tetrahedron at 1ms intervals. The distribution per tetrahedron is then expected to follow a binomial distribution, based on the reduced volume (the volume of the tetrahedron that is not occupied by vesicles). For this reason, the reduced volume was recorded per tetrahedron also at 1ms intervals.
Supplementary Fig. 4a shows the resulting distribution of molecules for 2 example tetrahedrons recorded from STEPS alongside the expected binomial distribution based on the mean reduced volume. Supplementary Fig. 4b displays the error in the binomial fit compared to the expected binomial for all 49 tetrahedrons, showing low error across all tetrahedrons. The reduced volume can be seen in Supplementary Fig. 4c for each tetrahedron.

Supplementary Note 13 | Mean First Passage Time of points undergoing 3D diffusion in a sphere

In this note, we consider a particle diffusing in 3D with diffusion coefficient *D* inside a sphere of radius *R*. We are interested in computing the mean first passage time (MFPT) of the particle to the surface of the sphere given that the point is initially uniformly distributed in the sphere. Considering only radial diffusion, the diffusion equation takes the form:

$$\frac{\partial C}{\partial t} = D\left(\frac{\partial^2 C}{\partial r^2} + \frac{2}{r}\frac{\partial C}{\partial r}\right) \quad (10)$$

We will consider that, once the particle reaches the surface of the sphere, it is moved back into the sphere with a uniformly distributed position. At steady-state we get:

$$D\left(\frac{\partial^2 C}{\partial r^2} + \frac{2}{r}\frac{\partial C}{\partial r}\right) + F = 0 \quad (11)$$

With *F* the constant flux representing particles reaching the boundary and being added back into the sphere. The resulting ODE is:

$$\frac{d}{dr}\left(r^2\frac{dC}{dr}\right) = -\frac{Fr^2}{D} \quad (12)$$

Which can be integrated to yield:

$$C(r) = -\frac{F}{6D}r^2 - \frac{A}{r} + B \quad (13)$$

with *A* and *B* being integration constants. The values of *A*, *B*, and *F* can then be determined by setting boundary conditions. The first one is the absorbing boundary condition at *r*=*R*:

$$C(R) = 0 \quad (14)$$

The second one ensures that only one particle is present in the sphere:

$$\int_0^R 4\pi r^2\, C(r)dr = 1 \quad (15)$$

The third one ensures that the flux of particles going out of the sphere matches the total flux with which particles go in:

$$\frac{4\pi}{3}R^3F = 4\pi R^2 j(R) \tag{16}$$

With *j(R)* the flux of particles reaching the surface of the sphere, determined from Fick's first law:

$$j(R) = -D\frac{dC}{dr}\bigg|_{r=R} \tag{17}$$

Applying the boundary conditions, we get:

$$C(r) = \frac{5}{2V}\left(1 - \frac{r^2}{R^2}\right) \tag{18}$$

With $V = \frac{4\pi}{3}R^3$ the volume of the sphere. We can then determine the average time τ taken by a point to exit the sphere by taking the inverse of the total flux of particles exiting the sphere (right hand side of equation 17). We get:

$$\tau = \frac{R^2}{15D} \tag{19}$$

Fig. 8c shows Supplementary Equation 19 alongside recorded error in second-order vesicle surface reaction rate when the cytosolic reactant is immobile. In addition, we also tested a model where the cytosolic reactant diffused at a rate of $1\mu m^2 s^{-1}$. Supplementary Fig. 5 shows the reduction in the error in second-order reaction rate, compared to Fig. 8c where the reactant is immobile, as expected.

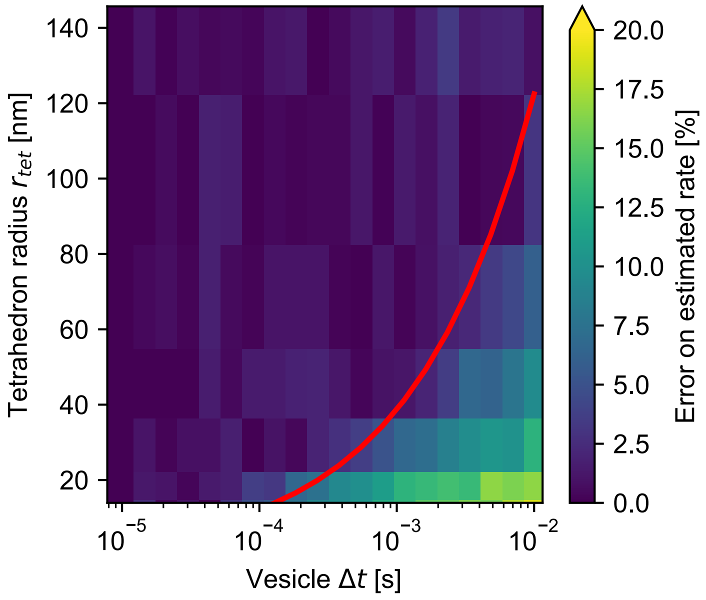

Supplementary Fig. 5 | Error on the rate of second-order vesicle surface reactions when a cytosolic reactant diffuses at $1\mu m^2 s^{-1}$. Red line depicts equation 19, as in Fig. 8c.

Supplementary References

[1] Rothman, J.S., Kocsis, L., Herzog, E., Nusser, Z., Silver, R.A.: Physical determinants of vesicle mobility and supply at a central synapse. Elife 5, 15133 (2016)
[2] Ghosh, A., Samuel, J., Sinha, S.: A "gaussian" for diffusion on the sphere. EPL (Europhysics Letters) 98(3), 30003 (2012)
