# Supplementary material for: Vesicle and reaction-diffusion hybrid modeling with STEPS
Source: Commun Biol. 2024 May 15;7:573. doi: 10.1038/s42003-024-06276-5 (PMC11096338; doi:10.1038/s42003-024-06276-5)
Supplement: Supplementary file 3 — Description of additional supplementary files [file 42003_2024_6276_MOESM3_ESM.pdf]

Description of Additional Supplementary Files

File name: Supplementary Movie 1

Description: Vesicles undergoing active transport on a path (not visualized) in STEPS, exhibiting dwelltimes sampled from an exponential distribution.

File name: Supplementary Movie 2

Description: An exocytosis model in STEPS, described further in text and extracted from [35]. The video shows calcium release in the cytosol, priming of the SNARE complexes, exocytosis and release of neurotransmitter into the extracellular space. Cell surface molecules (the remaining SNARE complex after exocytosis is complete) are not visualized.

File name: Supplementary Movie 3

Description: A model of vesicle clustering by synapsin dimer formation (although synapsin is not visualized). 300 vesicles are bound together but exhibit some reduced mobility within the cluster. 10 vesicles that do not interact with the cluster but do mix with it freely can be seen in green.
